# Diagnosis of cancer in the Emergency Department: A scoping review

**DOI:** 10.1002/cam4.5600

**Published:** 2023-01-09

**Authors:** Alix Delamare Fauvel, Jason J. Bischof, Raquel E. Reinbolt, Veronica K. Weihing, Edward W. Boyer, Jeffrey M. Caterino, Henry E. Wang

**Affiliations:** ^1^ Department of Emergency Medicine The Ohio State University Columbus Ohio USA; ^2^ Emergency Department Rouen University Hospital Rouen France; ^3^ Department of Internal Medicine The Ohio State University Columbus Ohio USA; ^4^ McGovern Medical School The University of Texas Health Science Center at Houston Houston Texas USA

**Keywords:** cancer diagnosis, diagnostic pathways, emergency department, emergency presentation, incidental diagnosis

## Abstract

**Background:**

The Emergency Department (ED) plays a key role in the identification and care of acute medical conditions, including cancer. In this scoping review, we aimed to determine the role of the ED in the acute diagnosis of cancer.

**Methods:**

We conducted a scoping review of articles according to Preferred Reporting Items for Systematic Review and Meta‐Analyses (PRISMA) using PubMed and Google Scholar. We screened studies of adults with a new diagnosis of cancer in the ED. We included randomized control trials, prospective, retrospective, and cross‐sectional observational studies, and case reports published in English since 2000. We grouped the articles into categories based on their objectives and findings.

**Results:**

Of the 4459 articles, we included 47 in the review. The identified studies fell into three major categories: (1) studies describing the incidental diagnosis of cancer in the ED (*n* = 11, 23%), (2) studies characterizing the acute initial presentation of cancer in the ED (*n* = 19, 41%), and (3) studies describing the ED as a pathway to cancer diagnosis in the healthcare system (*n* = 17, 36%). Across the studies, cancer diagnoses in the ED were more likely in patients with higher comorbidities, occurred at later stages, and resulted in worse survival rates.

**Conclusions:**

The ED plays a prominent role in the initial diagnosis of cancer. Efforts must be made to integrate the ED within the cancer care continuum.

**Summary**

The Emergency Department (ED) plays a key role in the diagnosis and care of acute medical conditions. The role of the ED in cancer diagnosis and management, however, is not well characterized. In this scoping review, we aimed to determine the role of the ED in the diagnosis of cancer. The prominent role played by the ED in the initial diagnosis and management of cancer argues that efforts must be made to integrate acute emergency care within the cancer care continuum.

**Statement**

The Emergency Department (ED) plays a prominent role in the initial diagnosis of cancer. Efforts must be made to integrate acute ED care within the cancer care continuum.

## 1 INTRODUCTION

Cancer is a major international public health issue.^1^, ^2^, ^3^ In the United States, there are 1.7 M new cases of cancer, resulting in almost 600,000 death each year.^4^, ^5^ The International Agency for Research on Cancer reported 18 million new cancer cases and 9.6 million cancer deaths worldwide in 2018.^6^, ^7^

The Emergency Department (ED) plays an important part in the delivery of emergency care for patients with cancer, including the identification of cancer, symptomatic treatment of cancer‐related complications, and the management of its related and antineoplastic treatment sequelae.^8^, ^9^, ^10^ In a cross‐sectional analysis of nationwide ED Sample data from 2006 to 2012, Rivera et al. estimated that there were over 4 million adult cancer‐related ED visits each year in the United States with an observed increase in visits each year.^11^ For instance, 20%–50% of the most common cancers (breast, colon, and lung) are diagnosed in an ED.^11^, ^12^, ^13^, ^14^

The National Cancer Institute has recognized that improved integration of the ED into the continuum of cancer care could lead to better patient management and outcomes.^15^ However, there are only limited perspectives on the role of the ED in cancer care. The optimal community management of cancer requires a system‐wide approach, encompassing integration of screening, detection, treatment, and recovery efforts. An enhanced understanding of the current roles of the ED in cancer care is potentially important, identifying opportunities for strengthening the integration of the ED in the continuum of cancer care. In this scoping review, we sought to characterize the role of the ED in cancer diagnosis.

## 2 MATERIALS AND METHODS

### 2.1 Protocol

We conducted a scoping review following the guidelines of the Preferred Reporting Items for Systematic reviews and Meta‐Analyses extension for Scoping Reviews (PRISMA‐ScR).^16^ (Table S1) Protocol registration at PROSPERO (International Prospective Register of Systematic Reviews) is not required for scoping reviews.

### 2.2 Search strategy

To identify relevant articles, we used the PubMed and Google Scholar databases. The search strategy is summarized in Table 1. We included original research published in 2000 or after involving adults >18 years old with new cancers diagnosed in the ED. We included randomized controlled trials, non‐randomized trials, prospective, retrospective, cross‐sectional studies, and case reports. We excluded non‐English manuscripts, reviews, studies dated before 2000, pediatric populations, studies of cancer diagnosed prior the ED visit, and animal studies. We used the web‐based Covidence software (*Veritas Health Innovation*, Melbourne, Australia) to manage the articles in the review.^17^

**TABLE 1:** Search strategy keywords

1	Cancer OR neoplasm OR neoplasia OR malignancy OR tumor
2	Diagnosis OR diagnose OR finding
3	Incidental OR unexpected OR surprise diagnosis
4	Emergency presentation OR presentation as an emergency
5	Emergency Department OR Emergency Room OR Emergency Units OR Emergency Service OR Emergency Ward
6	Diagnostic pathways
7	1 AND 3
8	1 AND 4
9	1 AND 6
10	1 AND 2 AND 3
11	1 AND 2 AND 4
12	1 AND 2 AND 6
13	1 AND 2 AND 3 AND 5
14	1 AND 2 AND 4 AND 5
15	1 AND 2 AND 5 AND 6

### 2.3 Study selection

A reviewer (ADF) performed an initial screening with manual searches involving the title and abstract evaluation and using keywords summarized in the search strategy (Table 1). We imported all identified articles into Covidence to remove duplicates, assess inclusion criteria, and facilitate data extraction. Two authors (ADF, VKW) used the title, abstract, and full text to confirm inclusion in the review. We required articles to include the keywords “Emergency Department,” “Emergency Room,” or any other related term usually used in the country of origin (for example, “Accident and Emergency” [A&E] Department in the United Kingdom), and “cancer” or its synonyms such as “neoplasm” or “neoplasia” or “malignancy” or “tumor.” We resolved discrepancies by consensus between the reviewing authors and the study team.

### 2.4 Data extraction and analysis

We extracted the following data elements from each study: title, first author, year, country of origin, type of cancer, aim, study design, study period, population (inclusion and exclusion criteria, number of participants), intervention, comparison/control, outcome(s), and primary results. We grouped the papers into categories based on findings and main themes outlined within the article to highlight the major results. Consistent with PRISMA guidelines for scoping reviews, we did not assess the risk of bias.^16^

## 3 RESULTS

### 3.1 Overview of the studies

**FIGURE 1:**
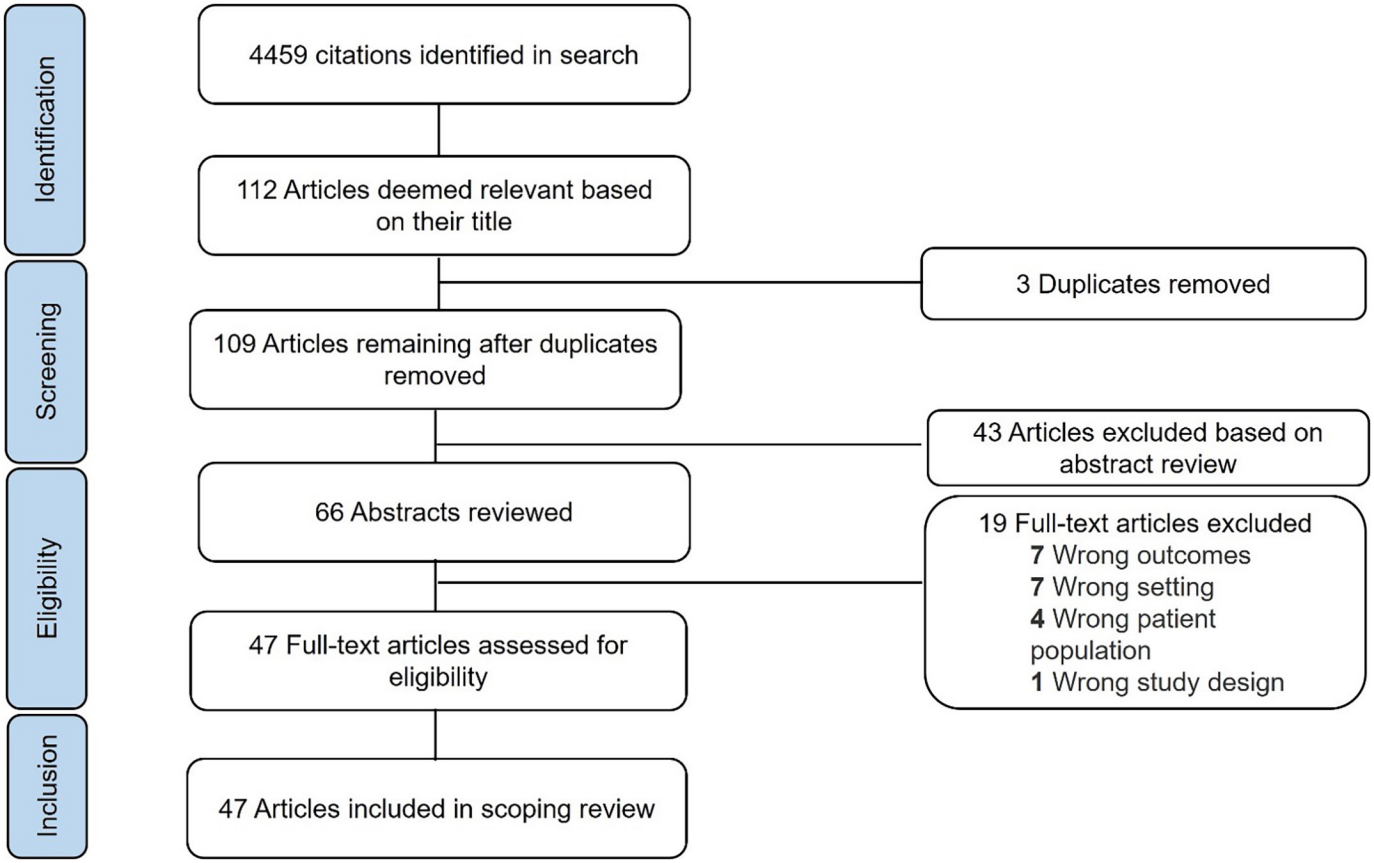
Preferred reporting items for systematic reviews and meta‐analyses (PRISMA) review flow diagram.

Of 4459 articles initially identified from PubMed and Google Scholar, we screened 112 full‐text manuscripts. We excluded 43 articles that did not meet the inclusion criteria. We excluded an additional 19 articles due to wrong outcomes (*n* = 7), wrong setting (*n* = 7), wrong patient population (*n* = 4), or wrong study design (*n* = 1). We included 47 articles in the final review (Figure 1).

**FIGURE 2:**
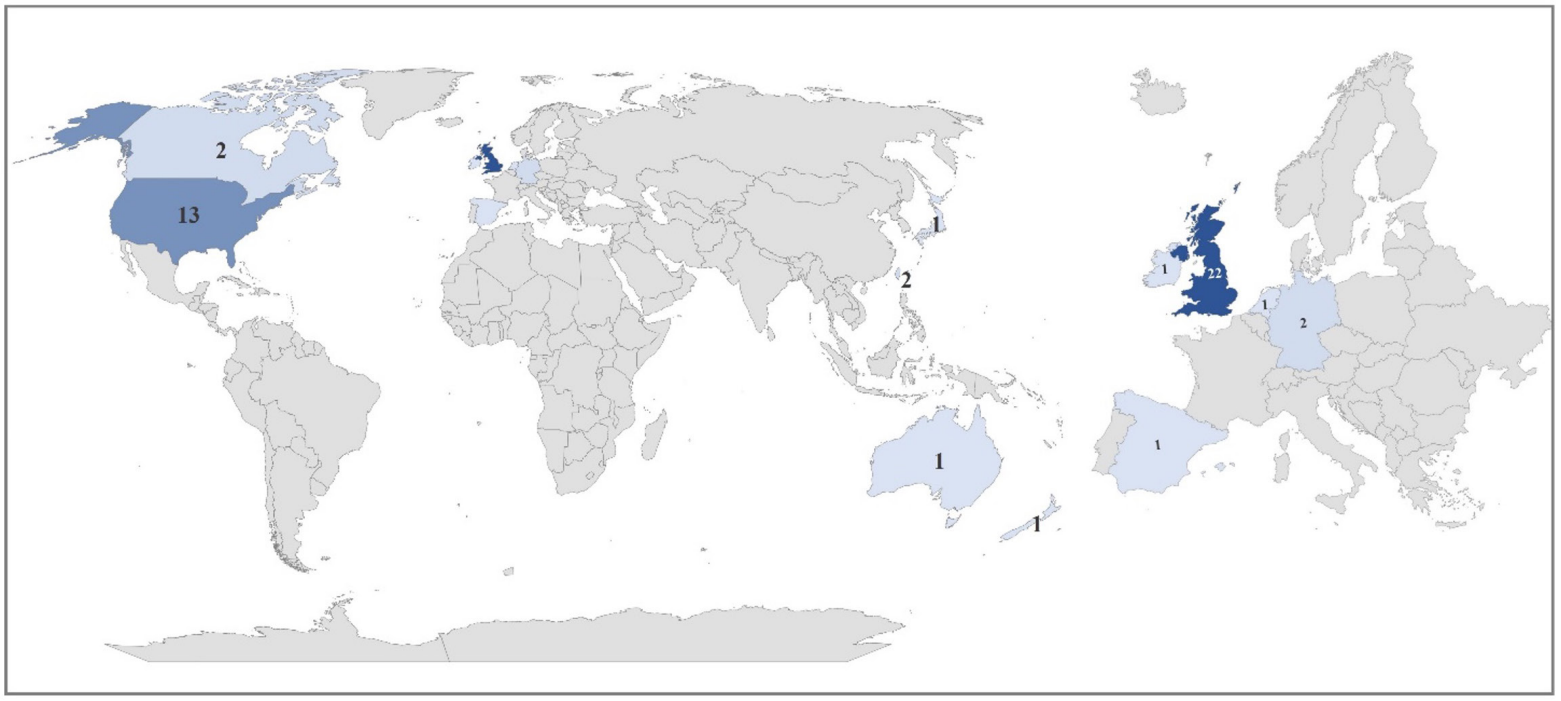
Origin of articles included.

The included studies were published between 2006 and 2021. Most studies originated from the United Kingdom (*n* = 22, 46.8%) or the United States (*n* = 13, 27.7%) (Figure 2). The types of cancers studied in the articles are described in Figure S1. The studies included mostly retrospective cohort (*n* = 32) and cross‐sectional (*n* = 10) designs as well as one prospective cohort, one case series, one qualitative study, one case–control study, and one randomized controlled trial. We identified three major themes among the included studies: (1) studies describing the incidental presentation of cancer diagnosis in the ED, (2) studies describing the acute initial presentation of cancer diagnosis in the ED, and (3) studies describing the ED as a pathway to cancer diagnosis in the healthcare system.

### 3.2 Incidental diagnosis of cancer in the Emergency Department

Cancer may be identified incidentally during the ED workup for other conditions; for example, cancerous conditions are often identified from computer tomography “pan‐scans” performed for major trauma. We found 11 studies describing cancers incidentally identified during the ED workup for other conditions, including 5 retrospective studies, 4 cross‐sectional studies, 1 randomized control trial, and 1 case–control study. Most articles originated from the United States (*n* = 5, 45.5%) with the remainder from Germany, Ireland, Netherlands, and the United Kingdom. The study populations ranged from *n* = 480 to 13,810. Ten of 11 articles referred to incidental diagnoses noted on CT scans; 7 involved CT scans for trauma.

**TABLE 2:** Studies describing incidental cancer diagnosis in the Emergency Department.

Reference	Country	Design	Population sample	Cancer type	Comparison	Outcomes	Main results
Hall^19^	United States	Cross‐sectional study	589	Thoracic	N/A	Prevalence of pulmonary embolism and other findings	Of CTAs, 24% had a new IF that required diagnostic follow‐up. Using current clinical guidelines, a follow‐up would be recommended for 96% of patients with new incidental pulmonary nodules
Munk^20^	United States	Retrospective cohort study	480	All cancers	N/A	IFs and their follow‐up in the discharge summary	IFs were noted in 43% of patients and on 15% of the CT studies performed for the patients. Most concerning lesions, such as suspected malignancies, accounted for 15% of all IF and were referred for follow‐up in only 49% of cases
Thompson^21^	United States	Retrospective study	682	All cancers	N/A	IFs	There were 682 CT scans among 600 patients: 199 Abdomen & Pelvis, 405 Head, and 78 Thorax. IFs were documented in 33.4% of the scans. Patients with one IF were less likely to receive disclosure than patients with two or more. Patients aged <60 were less likely to have IFs. There was no significant IF difference by gender
Fakler^67^	Germany	Retrospective cohort study	534	All cancers	N/A	IFs	Potentially life‐threatening, IFs were found in approximately every 15th patient, predominantly aged over 40 years, and presenting with minor to moderate injuries and an Injury Severity Score of 10 or less.
Kelly^22^	Ireland	Cross‐sectional study	1155	All cancers	N/A	IFs	On the 1155 emergency abdominal CT scan, 700 IFs were detected. Of the IF, 143 were deemed indeterminate requiring urgent investigations, and 24 occult neoplasms were confirmed subsequently
Kroczek^23^	Germany	Retrospective cohort study	2440	All cancers	N/A	IFs	Of IF found, 8.4% had an IF of high urgency (mostly indicative of malignancy or inflammation), and further 31.4% had IFs requiring follow‐up soon. Most IFs were seen in the abdomen/pelvis, followed by the thorax. There was a significant increase in the number and severity of IFs with the age of the patient.
Treskes^68^	Netherlands	Randomized controlled trial	1083	All cancers	TBTC scanning/selective CT scanning	IFs	TBTC scanning in trauma results in 1.5 times more IF. Evaluation by TBCT in trauma results in more patients with IFs
Mortani Barbosa Jr^69^	United States	Retrospective cohort study	1113	All cancers	N/A	Prevalence of traumatic injuries and IFs	IF are more likely to be found in a chest CT scan than acute traumatic injuries in patients being evaluated for potential traumatic injuries. Lung nodules were the most common IF. Radiologists recommended follow‐up for IF 53.5% of the time, but only 13.9% of patients ever received a follow‐up imaging exam or invasive procedure
Bell^24^	United States	Case–Control Study	670	All cancers	Control matched on age, sex, cancer site, cancer stage, year of diagnosis	30 days‐survival, cancer as cause of death	Long‐term mortality risks among trauma patients with incidental cancer diagnoses are no different than the cancer population among patients who survive at least 30 days after injury.
Koo^70^	United Kingdom	Cross‐sectional study	13,810	All cancers	Incidentally diagnoses/non‐incidentally diagnoses	Common pathways that led to the incidental diagnosis of cancer	Among the studied cancer patient population, 4% of patients were diagnosed incidentally. The odds of incidental cancer diagnosis increased with age, with no difference between men and women after adjustment
Lai^18^	Taiwan	Cross‐sectional study	4092	All cancers	N/A	IFs	IFs were identified in 15.9% of the scans enrolled. Old age remains the risk predicting the presence of IF, and every year of increasing age was independently associated with a higher prevalence of IFs. No sex‐based difference was found. Most of the scans were performed for the head; however, the abdomen had the highest IF prevalence

Abbreviations: CT, Computed Tomography; CTA, Computed Tomography Angiograms; ED, Emergency Department; IF, Incidental Finding; N/A, Not Available; TBTC, Total body computed tomography; United Kingdom, The United Kingdom; United States, The United States of America.

**TABLE 3:** Studies describing acute initial presentations of cancer

Reference	Country	Design	Population sample	Cancer type	Comparison	Outcomes	Main results
Barrett^33^	United Kingdom	Retrospective cohort study	151	CRC	No control group	Time intervals, pathways from the first consultation to the first referral	In this study, 26% were admitted through an emergency admission; 7% occurred after referral to a specialist but before the establishment of a diagnosis
Neal^71^	United Kingdom	Retrospective cohort study	889	Lung CRC Prostate Ovarian	Urgent referral (2WW)/Other routes including emergency referral	Survival, stage at diagnosis, delays in diagnosis, and secondary care delays	For lung cancer, urgent referrals had more advanced TNM stage and poorer survival than patients referred through other routes. There was no difference in stage or survival for CRC, prostate, or ovarian cancer
Elliss‐Brookes^25^	United Kingdom	Retrospective cohort study	739,667	All cancers	Eight routes of cancer diagnosis	One‐year relative survival	Eight routes are used to categorize all tumors (Screen Detected, 2WW, Emergency Presentation, GP Referral, Inpatient Elective, Other Outpatient, DCO, Unknown). Patients presenting via Emergency routes (24%, varying with cancer type) have substantially lower 1‐year relative survival
McPhail^34^	United Kingdom	Retrospective cohort study	131,754	Cervical CRC Breast Lung Prostate	No control group	Factors predictive of excess mortality rate after diagnosis	EP is highly predictive of cancer mortality in the year following diagnosis, and especially in the month following diagnosis. Excess mortality was strongly associated with EP, independently of case‐mix factors, disproving the hypothesis that it is because of these factors alone
Tsang^26^	United Kingdom	Cross‐sectional study	5870	All cancers	EP/other routes	Risk factors for diagnosis by emergency admission	Diagnosis by emergency was recorded in 13.9% of patients diagnosed with cancer for the first time. Patients of older age, living in the most deprived areas, or who had a total Charlson score of one compared to zero were most at risk of diagnosis by EP
Sheringham^35^	United Kingdom	Retrospective cohort study	943	CRC	“Emergency” versus “Other route” (i.e., GP and Consultant/ Other/ Unknown combined)	Rate of health service use in the 21 months before the diagnosis	CRC patients diagnosed through emergency routes (24%) tended to use primary care less and urgent care more frequently
Tataru^39^	United Kingdom	Retrospective cohort study	93,783	Lung	EP/non‐EP	Survival by route to diagnosis	Among all other lung cancer patients, those diagnosed through an emergency route had lower survival, particularly in the short‐term, but the association remained in the intermediate and long term
Abel^27^	United Kingdom	Retrospective Cohort Study	749,645	All cancers	No control group	Proportion of EP by sex, deprivation group, and age	Compared with men, women were at greater risk for EP in the bladder, brain, rectal, liver, stomach, colon, and lung, whereas the opposite was true for oral/oropharyngeal cancer, lymphomas, and melanoma. Similarly, younger patients were at higher risk for EP for acute leukemia, colon, stomach, and esophageal cancer, and older patients for laryngeal, melanoma, thyroid, oral, and Hodgkin's
Renzi^36^	United Kingdom	Retrospective cohort study	1606	CRC	Diagnosis as an EP/ Diagnosis as non‐EP	Signs and symptoms recorded in primary care before the cancer diagnosis	Emergency diagnosis occurred in 35% and 15% of the colon and rectal cancers. “Background” primary care consultations (2–5 years before diagnosis) were similar for either group. “Alarm” symptoms were recorded less frequently in emergency presenters
Abel^29^	United Kingdom	Retrospective cohort study	4647	All cancers	Prior consultation/ no prior GP consultation status	Prior GP consultation status and “three or more consultations” among prior consultees	Among emergency presenters, 29% reported no prior consultations, being more common in males, older, and the most deprived patients; and highest/lowest for patients with brain cancer and mesothelioma
Murchie^28^	United Kingdom	Retrospective cohort study	1802	All cancers	EP/non‐EP	EP	EP equaled 20% and 28% had no relevant prior GP contact. Associated predictors were no prior GP contact; having lung, colorectal and upper GI cancer; ethnicity
Maringe^40^	United Kingdom	Retrospective cohort study	264,813	Lung	Three types of EP: patient‐led, GP‐led, and “other”	GP characteristics and socio‐demographic characteristics of patients	Most EP reflect those patients by‐pass primary care. There are no GP characteristics predictive of unexpectedly high or low levels of EP
Renzi^37^	United Kingdom	Retrospective cohort study	5745	CRC	EP/non‐EP	Factors associated with EP diagnosis	CRC was diagnosed following EP in 34% of women and 30% of men. EP was more likely in women, independently of socio‐demographic factors and symptoms
Herbert^30^	United Kingdom	Retrospective cohort study	554,621	All cancers	GP referral/ Presentation to ED / Other EP sub routes	EP sub‐routes: ED referral, other EP	Among patients presenting as EP, 62% presented as ED referral. Patients presenting as EP were more likely to have been GP‐referred if they lived in less deprived areas or were subsequently diagnosed with pancreatic, gallbladder, ovarian cancer, or acute leukemia. During the study period, the proportion and number of ED referrals nearly increased
Herbert^31^	United Kingdom	Retrospective cohort study	2,042,192	All cancers	Proportions of EP in 2006/ proportions of EP in 2013	Time trends in the proportion of cancers diagnosed through EP.	The proportion of cancer diagnoses through EP is decreasing but age and deprivation inequalities prevail
Herbert^32^	United Kingdom	Retrospective cohort study	2,641,428	All cancers	No control group	EP status, independent main effect variables: diagnosis year and all four case‐mix variables	Between 2006 and 2015 there was an absolute 4.7 percentage point reduction in EP. Changes in case mix (particularly that of cancer sites) account for about a fifth of the overall reduction in emergency presentations
Joyce^72^	United Kingdom	Cross‐sectional study	6659	All cancers	No control group	Practice level factors	Practices in more deprived areas are significantly associated with a higher proportion of emergency diagnoses of cancer
Bright^38^	United Kingdom	Cross‐sectional study	69,178.	Lung CRC	No control group	EP by deprivation /age/sex/ethnicity group	Greater levels of deprivation were strongly associated with greater odds of EP, even after adjustment for the Mosaic group, which nonetheless attenuated. Similar findings were observed for increasing age
Athey^73^	United Kingdom	Retrospective cohort study	147	Ovarian	EP/Referral to a diagnostic clinic	Stage of disease at diagnosis	There was a significant association between later‐stage disease and EP and between increased age and later‐stage diagnosis. There was no association between stage at diagnosis and socioeconomic status

Abbreviations: CRC, Colorectal cancer; DCO, Death Certificate Only; ED, Emergency Department; EP, Emergency Presentation; GI, Gastro‐Intestinal; GP, General Practitioner; N/A, Not Available; TNM, tumor, node, metastasis; United Kingdom, The United Kingdom.

Incidental findings fell into three categories: (1) potentially serious conditions that need timely diagnostic work‐ups, (2) findings requiring consultation with other specialties and active management, and (3) non‐emergent findings.^18^

Several studies identified demographic and clinical occurrences of incidental findings on CT scans. The percentage of incidental findings on CT scans performed ranged from 15.9 to 43%.^18^, ^19^, ^20^, ^21^ The rate of occult malignancy diagnosis on CT scans ranged from 3.4 to 15%.^20^, ^21^, ^22^ In their study of 682 CT scans and 600 patients, Thompson et al. suggested that patients aged under 60 were less likely to have incidental findings, but there were no differences by gender.^21^ Incidental findings were more likely to be diagnosed on an abdominal CT than other parts of the body.^20^, ^21^, ^23^

Several studies described the subsequent course and the outcomes of incidental cancer findings. In a retrospective study of 480 patients, Munk et al. found that only 27% of discharge summaries mentioned the incidental findings in the discharge summary, documented an in‐hospital workup, or noted referral for outpatient follow‐up; only one‐half of suspected malignancies were referred for follow‐up.^20^ In a study of 134 trauma patients with incidental cancer diagnoses matched with 536 patients with cancer (controls), Bell et al. found that long‐term outcomes did not differ (Table 2).^24^

### 3.3 Acute initial presentation of cancer

Elliss‐Brookes et al. identified eight healthcare system pathways taken by patients with new cancer diagnoses: (1) screen‐detected, (2) the “Two‐Week Wait,” (3) general practitioner referral, (4) other outpatients, (5) inpatient elective, (6) emergency presentation, (7) death certificate only, and (8) unknown. Many cancer patients present acutely at diagnosis and require urgent care, defined by an ED visit, an emergency hospital admission or transfer, or an emergency consultant outpatient referral.^25^

We identified 19 studies describing the acute initial presentation of cancer, including retrospective cohort (*n* = 16, 84%) and cross‐sectional studies (*n* = 3, 16%). All the studies were originated from the United Kingdom. The study sample sizes ranged from *n* = 147 to 2,641,428. The cancers encompassed all types but were mainly lung and colorectal. The percentage of acute cancers requiring urgent care as defined above ranged from 13.9 to 31% across cancer types.^25^, ^26^, ^27^, ^28^, ^29^, ^30^, ^31^, ^32^ Acute initial presentations ranged from 23 to 35% for colorectal cancer^33^, ^34^, ^35^, ^36^, ^37^, ^38^ and from 24 to 37.5% for lung cancer.^34^, ^38^, ^39^, ^40^

Three major studies examined the risk factors for the acute initial presentation of cancer. Using a cohort of 131,754 patients with cervical, colorectal, breast, lung, and prostate cancer, McPhail et al. found that older age, comorbidities, and income deprivation were associated with acute initial presentations.^25^, ^34^ In a population of 5870 patients, Tsang et al. found that the breast and the colorectal cancer were most likely to present acutely.^26^ In a cohort of 749,645 patients, Abel et al. observed brain cancer and acute lymphocytic leukemia were most likely to result in acute initial presentations.^27^

Several studies described the outcomes of patients with the acute initial presentation of cancer. These studies suggest that acute initial presentation is associated with later‐stage cancer and increased mortality in the year following diagnosis. The difference in survival between acute and non‐acute presentations ranges between 20 and 40%.^25^, ^34^ In a study of 93,783 patients with lung cancer, Tataru et al. found that those diagnosed through an emergency route had lower short‐term (adjusted HR = 3.54, 95% CI: 3.42–3.67), intermediate (adjusted HR = 1.66, 95% CI: 1.63–1.69), and long‐term (adjusted HR = 1.10, 95% CI: 1.05–1.15) survival (Table 3).^39^

### 3.4 The Emergency Department as a pathway to cancer diagnosis

Cancer patients often connect with oncology care through different routes in the healthcare system. We identified 17 studies that contrasted cancer diagnosed in the ED versus other healthcare settings. There were 11 retrospective cohorts, 1 prospective cohort, 3 cross‐sectional studies, 1 qualitative study, and 1 case series. Almost half of those originated in the United States (*n* = 8, 47.1%). Other studies came from the United Kingdom, New Zealand, Taiwan, Japan, Australia, Canada, and Spain. Sample sizes ranged from *n* = 6 to 20,311. Two‐thirds of the studies involved lung and/or colorectal cancers (*n* = 11, 64.7%). The studies found that 12–32% of cancer diagnoses occurred through the ED.^14^, ^41^ Cancer diagnosis pathways varied by cancer type; for example, lung cancers diagnosed through an ED ranged from 10 to 57%^14^, ^42^, ^43^, ^44^, ^45^, ^46^ while colorectal cancer diagnosed through an ED ranged from 15 to 42%^14^, ^47^, ^48^, ^49^ (Table 4).

**TABLE 4:** Studies describing the emergency department as a pathway to cancer diagnosis

Reference	Country	Design	Population sample	Cancer type	Comparison	Outcomes	Main findings
Mitchell^74^	Canada	Prospective cohort study	455	CRC	ED patients/Elective resection	Length of stay, perioperative mortality	The ED cohort (24% of the study population) was older, counted more females, and was associated with a more advanced TNM stage, a longer length of stay in the hospital, and higher perioperative mortality
Beatty^42^	New Zealand	Retrospective cohort study	478	Lung	Cancers presented via the ED / Cancers presented via other routes	Previous contact with the health system before ED presentation, presenting symptoms	Of lung cancer cases, 36% presented via the ED. ED presentation varied with tumor stage, ethnicity, and District Health Board. Age, gender, and tumor type were not associated with ED presentation
Sikka^47^	United States	Retrospective cohort study	20,311	CRC Lung	Diagnosis associated with an ED visit/Diagnosis not associated with an ED visit	ED use 3–12 months before cancer diagnosis, stage at diagnosis	Patients with a CRC or lung cancer diagnosis associated with an ED visit had more comorbidities, were more likely to be female, older, and diagnosed at a later stage compared with patients diagnosed in other settings
Chen^48^	Taiwan	Retrospective cohort study	154	CRC	Patients referred from the ED/patients referred from non‐ED sources	Staging at diagnosis, Survival at 2 years	Patients comprised the ED group (29%) had significantly longer hospital stays and greater in‐hospital mortality but there was no statistically significant difference in stage at diagnosis or 2‐year mortality rate between the groups
^a^Amri^46^	United States	Retrospective cohort study	1071	Colon	Patients diagnosed in the ED/Elective presentation	Overall survival, Disease‐free survival	Patients diagnosed in the ED required longer surgeries, longer median admissions, more readmission, and perioperative mortality. Adjusting for staging, ED patients had higher mortality and shorter disease‐free survival
^a^Fujimoto^43^	Japan	Retrospective cohort study	771	Lung	Diagnosis following emergency admission / Non‐diagnosis following emergency admission	Survival	Diagnosis following admission via the ED (13% of patients) was not an independent predictor of overall survival
Black^75^	United Kingdom	Qualitative study	27	All cancers	No control group	Patients' experiences of their diagnosis in an ED	The typical experience of patients diagnosed through an ED diverges significantly from normative pathways even when there is no evidence of serious service failures
Rogers^14^	Australia	Cross‐sectional	1307	All cancers	ED visits leading to cancer diagnosis/ED visits not leading to cancer diagnosis	Survival	One in 10 newly diagnosed cancer patients was diagnosed as a result of an ED visit. These patients were older, more often men, from disadvantaged areas with advanced stage and shorter survival
Livingood^41^	United States	Retrospective cohort study	989	All cancers	ED‐associated initial cancer diagnoses / non–ED associated initial diagnoses	Stage of cancer and death. Discharge, destination, hospital length of stay, and total inpatient charges	One‐third of initial cancer diagnoses patients were admitted via ED with disproportionately higher rates for African Americans and impoverished, more advanced higher stage, and increased risk of death
^a^Esteva^76^	Spain	Cross‐sectional study	950	CRC	ED/Presentation to outpatient services	Factors associated with diagnosis in the ED of CRC patients	Approximately 40% of CRC patients first contacted a hospital for CRC through an ED. Women were more likely than men to be emergency presenters. Lack of contact with a GP for CRC‐related symptoms was related to ED diagnosis
^a^Solsky^77^	United States	Retrospective cohort study	263	Gastric	ED‐associated initial cancer diagnoses / non–ED associated initial diagnoses	Survival	ED‐diagnosed gastric cancer patients (52% of patients) were older, had more comorbidities, and increased mortality
^a^Salika^78^	United Kingdom	Retrospective Cohort Study	6837	CRC	Routes to diagnosis	Aspects of the patient journey experience from diagnosis to post‐treatment care	Screening‐detected cancer patients reported the best care. ED‐detected cancer patients reported the worst care experience
Suhail^44^	Canada	Cross‐sectional study	951	Lung	Cancers diagnosed in the ED / Cancers non‐diagnosed in the ED	Disease stage, survival	ED lung cancer diagnosis (35% of patients) was associated with advanced stage and higher mortality
Yee^79^	United States	Retrospective cohort study	90	Breast	No control group	Demographics	Women diagnosed in the ED with breast cancers were most often Hispanics or African Americans, with a significant delay in follow‐up primary care visits
Weithorn^49^	United States	Retrospective cohort study	638	CRC	ED‐associated initial cancer diagnoses / non–ED associated initial diagnoses	Survival	CRC patients diagnosed in ED (42% of patients) were more likely to be older than 80 years, have Medicare or Medicaid, present with symptoms, advanced stage, and had increased mortality
Pettit^80^	United States	Case series	6	Lung	N/A	N/A	Patients diagnosed in the ED have complex care pathways including delayed biopsies, delayed treatments, and poor outcomes
Pettit^45^	United States	Retrospective cohort study	268	Lung	No control group	Cancer type, Stage	Lung cancers initially presented to the ED (57% of patients) were generally elderly, African American, and smokers, presenting with more advanced stage and higher mortality

Abbreviations: 2WW, two week‐wait; CRC, Colorectal cancer; DCO, Death Certificate Only; ED, Emergency Department; EP, Emergency presentation; GP, General Practitioner; TNM, tumor, node, metastasis; United Kingdom, The United Kingdom; United States, The United States of America.

^a^ Articles for which there may be an overlap between the category of ED as a pathway to cancer diagnosis and the category of acute initial presentations of cancer, or for which the term emergency is ambiguous as to whether it refers to the department or the presentation, or can evoke both at the same time.

In a study of 989 patients with varying types of cancer, Livingood et al. showed that patients diagnosed with cancer in the ED were more likely to be male (RR 1.29 95% CI [1.07–2.03]), African‐American (RR 1.46 95% CI [1.21–1.76]), and to have Medicaid, Medicare, or no health insurance (RR 2.67 95% CI [1.60–4.65]; RR 3.10 95% CI [1.87–5.39]; RR 4.35 [2.63–7.54]), respectively.^41^ In a study of 20,000 patients with colon or lung cancer, Sikka et al. found that compared with other settings, patients with a colorectal or a lung cancer diagnosis associated with an ED visit were more likely to have a higher comorbidity burden, to be female, to have an inpatient admission before diagnosis, and to be aged 80 years and older. Patients with an ED visit near a colorectal cancer or lung cancer diagnosis were more likely to be diagnosed at a later stage compared with patients diagnosed in other settings.^47^ Weithorn et al. reported on colorectal cancer ED‐associated initial diagnoses compared to non‐ED diagnoses and found that ED‐associated initial diagnoses were associated with increased mortality (HR: 1.89, 95% CI: 1.3–2.8), even when stratifying survival by stage.^49^ Sikka et al. and Weithorn et al. found that colorectal cancer patients diagnosed through the ED were older with more comorbidities.^47^, ^48^, ^49^ Weithorn et al. also showed that colorectal cancer patients diagnosed through the ED were more symptomatic than those through other settings (OR 6.93 95% CI [3.59–13.36]) Table 4.^49^

## 4 DISCUSSION

The ED plays a prominent role in cancer diagnosis. We identified three major themes characterizing the role of the ED: (1) many cancers are identified during the ED workup for unrelated complaints, most often as incidental findings on CT scans, (2) many cancers present as an acute condition that requires emergency care, and (3) the ED is frequently a pathway that accelerates healthcare system cancer diagnosis and care. Across all of these themes, ED cancer patients (compared with those receiving a diagnosis in other settings) have a higher comorbid burden, present with cancers at later stages, and have poorer outcomes. These studies underscore the importance of the ED in the continuum of cancer care.

Our findings highlight large gaps in knowledge in ED cancer diagnosis. Understanding how cancer patients use the ED as a pathway for cancer diagnosis is the first step to improving their care and their follow‐up. Systemwide studies are needed to further characterize the epidemiology of cancers diagnosed and treated in the ED. National series using the National Hospital Ambulatory Medical Care Survey and National Emergency Department Survey describe cancer patients seeking ED care but offer only limited perspectives of their course and outcomes.^8^ The relationship between substantial increased numbers of ED‐diagnosed cancers and the lack of access to primary care or cancer screening remains undefined. It is also unclear if system or care enhancements could improve the care or outcomes of cancer patients identified in the ED. For example, select centers in the United States have established specialized cancer EDs or urgent care centers with expertise in the management of acute cancers and the complications of chronic cancer treatment such as chemotherapeutic toxicity.^50^, ^51^, ^52^, ^53^ Strengthening the link between oncology and emergency medicine teams with a multidisciplinary approach could improve the quality, efficiency, and continuity of care for these patients.^10^, ^50^, ^54^, ^55^, ^56^ The Comprehensive Oncologic Emergencies Research Network (CONCERN) was established with support from the National Cancer Institute to expand the knowledge around the treatment of oncologic emergencies in the emergency medicine setting by facilitating collaborations across oncology and emergency medicine.^57^

Another essential knowledge gap highlighted by our study entails pathways of cancer care. As highlighted by Ellis‐Brookes, the linkage of cancer patients with network oncology care is a complex process, even for communities with sophisticated systems of cancer care.^25^ Factors influencing patient linkage with care may include patient geography, insurance status, baseline health functioning, and social support. Our review highlights that many cancer patients link to health system cancer care through the ED. While some of these cases may be unavoidable (for example, the patient with a colon tumor identified incidentally during evaluation of injuries from a motor vehicle collision), some patients may have missed opportunities for screening and earlier diagnosis. Our review also highlights that current systems of referral and linkage often do not meet clinical needs, with large portions of ED‐identified cancers having marginal or no follow‐up. These findings collectively suggest the need for standardization of referral and linkage processes. The COVID‐19 pandemic has further exacerbated this issue, with reports of a drastic worldwide reduction in rates of cancer screening.^58^ The creation of rapid cancer diagnostic centers may enhance testing and care access.^50^, ^56^

## 5 LIMITATIONS

Some limitations exist in this scoping review. We limited the review to articles written in English; most of the included articles originated from the United States and the United Kingdom and may not be generalizable worldwide. Healthcare systems in other countries vary. We would expect similar variations for cancer detection and care. Indeed, studies that have analyzed ED visits of oncologic patients vary depending on the geographical location of the study. Although the reasons for visiting are often the same (pain, nausea‐vomiting, or fever), the types of cancer, the anti‐cancer therapies used, and the percentage of home discharge vary greatly in Africa,^59^ Asia,^60^, ^61^ Europe,^62^, ^63^ and America.^64^, ^65^ Indeed, the epidemiology of cancers varies substantially vary across countries depending on the degree of economic development and associated social and lifestyle factors.^6^ Future study must spotlight the distinctions of cancer detection internationally.

There was variation in the definition of “emergency presentation” and “ED.” Zhou et al. noted a substantial degree of heterogeneity in how the diagnosis of cancer as an emergency was defined, with clinical criteria (whether life‐threatening symptoms were present or emergency treatment used) as opposed to contextual criteria (whether the patient presented to emergency services).^25^, ^66^ We tried to keep these two concepts separate, but a certain degree of overlap remains. We indicated these articles in Table 4. Consistent use of this term would be valuable for future research in this area.

## 6 CONCLUSIONS

This review suggests that the diagnosis of cancer in the ED, whether incidental or due to symptom burden, represents an increasingly important route of cancer diagnosis. Strengthening the link between oncology and emergency medicine teams with a multidisciplinary approach could improve the quality, efficiency, and continuity of care. Future research evaluating ED use, the impact of specialized cancer EDs on cancer diagnosis, and the trends and outcomes of incidental versus acute initial presentation of cancers diagnosed in the ED are needed to further clarify the current and the future role of the ED in the cancer care continuum.

## AUTHOR CONTRIBUTIONS

**Alix Delamare Fauvel:** Conceptualization (equal); data curation (equal); formal analysis (equal); writing – original draft (lead); writing – review and editing (equal). **Jason J Bischof:** Conceptualization (equal); validation (equal); writing – review and editing (equal). **Raquel Reinbolt:** Conceptualization (equal); validation (equal); writing – review and editing (equal). **Veronica Weihing:** Formal analysis (equal). **Edward Boyer:** Conceptualization (equal); funding acquisition (lead); writing – review and editing (equal). **Jeffrey M. Caterino:** Writing – review and editing (equal). **Henry Wang:** Conceptualization (lead); methodology (lead); validation (lead); writing – review and editing (lead).

## FUNDING INFORMATION

Dr. Boyer is supported by K24DA027109 and R01DA047236.

## CONFLICT OF INTEREST

The authors declare no conflict of interest.

## Supporting information


Figure S1.

Table S1.

## Data Availability

Data sharing is not applicable to this article as no new data were created or analyzed in this study.
