# Supplementary figures and images for: Diagnosis of cancer in the Emergency Department: A scoping review
Source: Cancer Med. 2023 Jan 9;12(7):8710–28. doi: 10.1002/cam4.5600 (PMC10134283; doi:10.1002/cam4.5600)

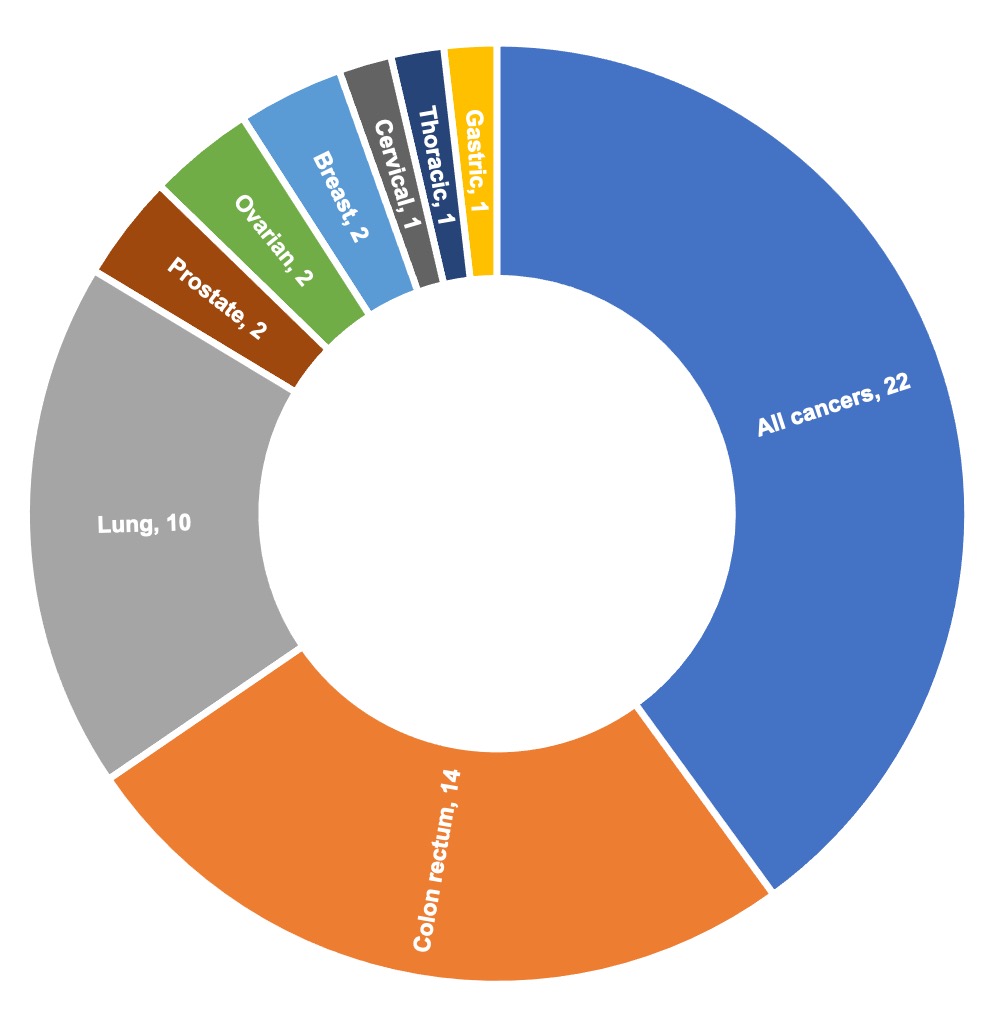

Supplement: Supplementary file 1 — Figure S1. [file CAM4-12-8710-s002.jpg]
